# Skeletal muscle ACC2 S212 phosphorylation is not required for the control of fatty acid oxidation during exercise

**DOI:** 10.14814/phy2.12444

**Published:** 2015-07-08

**Authors:** Hayley M O’Neill, James S Lally, Sandra Galic, Thomas Pulinilkunnil, Rebecca J Ford, Jason R B Dyck, Bryce J van Denderen, Bruce E Kemp, Gregory R Steinberg

**Affiliations:** 1 Division of Endocrinology and Metabolism, Department of Medicine, McMaster UniversityHamilton, Ontario, Canada; 2 Department of Medicine, St. Vincent’s Institute of Medical Research, University of MelbourneFitzroy, Victoria, Australia; 3 Faculty of Health Sciences and Medicine, Bond Institute of Health and Sport, Bond UniversityRobina, Queensland, Australia; 4 Department of Pediatrics, Faculty of Medicine and Dentistry, Cardiovascular Research Centre, Mazankowski Alberta Heart Institute, University of AlbertaEdmonton, Alberta, Canada

**Keywords:** Acetyl-CoA carboxylase 2, AMP-activated protein kinase, exercise, fatty acid/metabolism, knock-in mice, malonyl-CoA

## Abstract

During submaximal exercise fatty acids are a predominant energy source for muscle contractions. An important regulator of fatty acid oxidation is acetyl-CoA carboxylase (ACC), which exists as two isoforms (ACC1 and ACC2) with ACC2 predominating in skeletal muscle. Both ACC isoforms regulate malonyl-CoA production, an allosteric inhibitor of carnitine palmitoyltransferase 1 (CPT-1); the primary enzyme controlling fatty acyl-CoA flux into mitochondria for oxidation. AMP-activated protein kinase (AMPK) is a sensor of cellular energy status that is activated during exercise or by pharmacological agents such as metformin and AICAR. In resting muscle the activation of AMPK with AICAR leads to increased phosphorylation of ACC (S79 on ACC1 and S221 on ACC2), which reduces ACC activity and malonyl-CoA; effects associated with increased fatty acid oxidation. However, whether this pathway is vital for regulating skeletal muscle fatty acid oxidation during conditions of increased metabolic flux such as exercise/muscle contractions remains unknown. To examine this we characterized mice lacking AMPK phosphorylation sites on ACC2 (S212 in mice/S221 in humans-ACC2-knock-in [ACC2-KI]) or both ACC1 (S79) and ACC2 (S212) (ACC double knock-in [ACCD-KI]) during submaximal treadmill exercise and/or ex vivo muscle contractions. We find that surprisingly, ACC2-KI mice had normal exercise capacity and whole-body fatty acid oxidation during treadmill running despite elevated muscle ACC2 activity and malonyl-CoA. Similar results were observed in ACCD-KI mice. Fatty acid oxidation was also maintained in muscles from ACC2-KI mice contracted ex vivo. These findings indicate that pathways independent of ACC phosphorylation are important for regulating skeletal muscle fatty acid oxidation during exercise/muscle contractions.

## Introduction

The AMP-activated protein kinase (AMPK) is an evolutionarily conserved metabolic stress-sensing kinase that is rapidly activated in response to muscle contractions in both rodents and humans (for review see O’Neill 2013). In skeletal muscle the activation of AMPK is proposed to restore energy balance by switching on ATP-producing pathways such as glucose uptake and fatty acid oxidation. The role of AMPK in regulating skeletal muscle fatty acid oxidation has been studied intensively using the pharmacological agent 5-aminoimidazole-4-carboxamide ribonucleotide (AICAR) (Munday et al. 1988a,b; Merrill et al. 1997; Koistinen et al. 2003; Dzamko et al. 2008). AICAR increases skeletal muscle AMPK activity and reduces the activity of acetyl-CoA-carboxylase 2 (ACC2). Reductions in ACC2 activity are associated with lower muscle malonyl-CoA, which is an allosteric inhibitor of carnitine palmitoyl transferase (CPT)-I; the rate-limiting enzyme that controls the transfer of cytosolic long-chain fatty acyl-CoAs into the mitochondria for *β* oxidation (Winder and Hardie 1996; Rasmussen and Winder 1997). Consistent with an important role for ACC2 in regulating skeletal muscle fatty acid oxidation some (Abu-Elheiga et al. 2001; Hoehn et al. 2010) but not all (Olson et al. 2010) studies have shown that mice deficient in ACC2 have higher rates of skeletal muscle fatty acid oxidation. In agreement with an important role for ACC2 in skeletal muscle, mice with a targeted knock-in mutation to the AMPK phosphorylation site on ACC2 (S212 in mice which is the equivalent to S221 in humans) are insensitive to AICAR-stimulated increases in fatty acid oxidation (O’Neill et al. 2014).

Despite the compelling evidence indicating a vital role for AICAR to regulate skeletal muscle fatty acid oxidation via an AMPK-ACC2-dependent pathway, the importance of this pathway for increasing fatty acid oxidation during exercise/muscle contraction is not clear. For example, rates of fatty acid oxidation increase until ∼65% of maximal oxygen uptake (Jeukendrup 2002); however, AMPK and ACC phosphorylation are only partially increased at these low exercise intensities and malonyl-CoA content is unchanged or only modestly reduced (Odland et al. 1996; Roepstorff et al. 2005). In contrast, during high-intensity exercise, where carbohydrates are preferentially utilized and absolute rates of fatty acid oxidation actually decline (Romijn et al. 1995), AMPK is potently activated but malonyl-CoA levels do not change (Odland et al. 1998). Similarly, AMPK activity and ACC phosphorylation is reduced in trained versus untrained humans and rodents despite their greater utilization of fatty acids during exercise at the same absolute or relative workload (Durante et al. 2002; McConell et al. 2005). Consistent with a mismatch between AMPK activity and fatty acid oxidation during exercise/muscle contractions, mice that have reductions in skeletal muscle AMPK activity appear to have normal (Dzamko et al. 2008; Steinberg et al. 2010; Jeppesen et al. 2013) or slightly higher rates of fatty acid oxidation (O’Neill et al. 2011) during exercise; although it should be noted that a very recent report has found that AMPK *α* muscle null mice have a higher RER (indicative of lower whole-body fatty acid oxidation) during treadmill exercise and a modest reduction in contraction-stimulated fatty acid oxidation in soleus muscle (Fentz et al. 2015). Surprisingly, despite large reductions in muscle AMPK activity in all of the above mouse models there are increases in the phosphorylation of ACC2 during exercise/muscle contractions; thus making it difficult to determine the importance of ACC2 phosphorylation for controlling fatty acid oxidation.

Therefore, the purpose of this study was to assess fatty acid oxidation in mice lacking the AMPK phosphorylation site on ACC2 (ACC2-KI). To examine potential compensation by ACC1, which is expressed at extremely low levels in muscle but has been suggested to possibly compensate for a lack of ACC2 (Olson et al. 2010), we also studied mice lacking the AMPK phosphorylation site on both ACC1 and ACC2 (ACCD-KI). We hypothesized that during exercise/muscle contractions ACC2-KI and ACCD-KI mice would have impaired rates of fatty acid oxidation due to elevated ACC2 activity and malonyl-CoA, which would reduce endurance exercise capacity and/or muscle performance. Surprisingly, we found that while ACC2 S212 phosphorylation is important for reducing ACC2 activity and malonyl-CoA levels during exercise this appears to have little bearing on rates of skeletal muscle fatty acid oxidation, thus providing evidence that ACC2-independent pathways are capable of regulating fatty acid oxidation during muscle contractions.

## Methods

### Mice- environment and diet

ACC2 S212A KI (ACC2-KI) and ACC1/2 KI (ACC-DKI) mice have been recently described (Fullerton et al. 2013). Male mice were housed in Specific Pathogen Free microisolators and maintained under control environment conditions (12 h/12 h light–dark cycle with lights on at 07:00 and temperature of 23°C). Mice had ad libitum access to water and standard chow diet (17% kcal fat; Diet 8640, Harlan Teklad, Madison, WI) until experiments were completed between 10 and 16 weeks of age. All experiments were approved by the McMaster University and St. Vincent’s Hospital, Animal Ethics Committees.

### Blood analyses

Mice were exercised (60 min at 65% maximal running speed) before whole-blood samples were taken from tail blood at 0, 30, and 60 min during exercise, and placed on ice in tubes containing EDTA before centrifugation at 1830 *g* for 10 min at 4°C and collection of plasma. Plasma was stored at −80°C before use in analyses. Blood glucose was determined by glucometer (Accu-Check, Roche Diagnostics, Milpitas, Germany). Plasma nonesterified free fatty acids (NEFA) and lactate were assessed by colorimetric analysis using a nonesterified fatty acid (NEFA) (Wako Chemicals, Osaka, Japan) and L-lactate (Biovision, Milpitas, CA) kits, respectively, adapted for use in a 96 well microplate. Microplates were read using a Polarstar Optima microplate reader.

### Treadmill exercise

Prior to treadmill running experiments, mice were acclimatized as recently described (O’Neill et al. 2014). For maximal exercise capacity testing, mice ran at a 5 degree gradient at 10 m/min for 2 min before intensity (running speed) was increased by 1 m/min every 2 min until mice could not be prompted to continue running by bottle brushes and electric shockers at the back of the treadmill (i.e., “exhaustion”). For endurance capacity testing, mice were run at 65% of their maximal running capacity at a 5° inclination until exhaustion. The following week, VO_2_, VCO_2_, RER, and % substrate utilized were determined by running mice at 65% of maximal running speed in an enclosed Oxymax treadmill (Columbus Instruments, Columbus, OH) for 60 min. Total lipids oxidized during exercise were determined by calculating mean fatty acid oxidation rate ((1.6946*O_2_)−(1.7012*VCO_2_)) (mg/kg/h) (Frayn 1983).

### Palmitate oxidation in isolated muscles

Experiments were conducted as previously described (Dzamko et al. 2008; Steinberg et al. 2010). Briefly, muscles were transferred to organ baths (Radnoti, Monrovia, CA) filled with prewarmed (30°C) Krebs–Hanseleit basal buffer (NaCl, 119 mmol/L; KCl, 4.7 mmol/L; CaCl_2_, 2.5 mmol/L; MgSO_4_, 1.2 mmol/L; KH_2_PO_4_, 1.2 mmol/L; NaHCO_3_, 25 mmol/L) supplemented with pyruvate (2.0 mmol/L), fatty acid free BSA (4%), and palmitate (0.5 mmol/L) for 20 min. The distal tendon of the muscle was tied to a fixed, immovable hook and the proximal tendon was attached vertically to a force transducer (Model TRI202P, PanLab, Barcelona, Spain). Incubation chambers were oxygenated with 95% O_2_: 5% CO_2_ and thermostatically maintained at 30°C. Gas supply to the muscle was shut off and the buffer was replaced with a similar buffer (0.5 mmol/L palmitate) supplemented with [1-^14^C]-palmitic acid (0.5 *μ*Ci/mL for a specific activity of ∼830 dpm/nmol). One ml of mineral oil was placed on top of tracer buffer to prevent ^14^CO_2_ loss during contractions. The muscles were stimulated with a series of isometric twitch contractions, with muscle length adjusted between responses to determine the optimum length (Lo); the length at which twitch force was optimal. Muscles were stimulated (50 Hz, 40V, 600 ms pulse duration, 6 tetani/min) for 20 min. This contraction protocol has previously been shown to stimulate fatty acid oxidation maximally during contractions of isolated muscles (Dyck and Bonen 1998).

### Analytical methods

#### Western blotting

Muscles lysates were prepared and subjected to Western blotting as previously described (O’Neill et al. 2011). Protein phosphorylation and expression levels were determined by SDS-PAGE followed by immunoblotting using muscle lysates that were adjusted to equal protein concentration (2 *μ*g/*μ*L) and boiled for 5 min at 95°C in 4× sample buffer (Tris·HCl [50 mmol/L, pH 6.8], SDS [2%], glycerol [10%], DTT [1%], EDTA [1%], and bromophenol blue [0.02%]). Primary antibodies for determination of phosphorylation status and total expression of various proteins are as follows: AMPK phospho-*α*T172 (#2531; Cell Signaling Technology, Danvers, MA), ACC phospho-S79 (also detects S221 site of ACC2 [S212 in mouse]) (#3661; Cell Signaling Technology), CPT-1 (CPT1M11-A; Alpha Diagnostics, San Antonio, TX), sirtuin 3 (SIRT3) (#86671; AbCam, Cambridge, UK) and uncoupling protein 3 (UCP3) (#3477; AbCam). Glyceraldehyde 3-phosphate dehydrogenase (GAPDH) (#9483; AbCam), *α* actinin (# A7811; Sigma Aldrich, St Louis, MO), pan actin (#8456; Cell Signaling Technology) or voltage-dependent anion channel (#4866; Cell Signaling Technology) were used as a protein loading control or Steptavidin-HRP (#3999; Cell Signaling Technology) for total ACC. Membranes were washed 3 × 10 min with PBST and incubated with an appropriate HRP-conjugated secondary antibody (1:10,000) for 1 h at room temperature. Protein bands were visualized using a Fusion Image Dock Station (Vilber Lourmat, Eberhandzell, Germany) and enhanced chemiluminescence (ECL^+^). Bands were quantified using ImageJ software and protein content was expressed in relative units in comparison with control samples loaded on each gel or in the case of phosphorylated proteins as a ratio of the phosphorylated to total protein (e.g., ACC-p S79/212/ACC). Membranes used for detection of phosphorylated AMPK or ACC were stripped with a buffer containing 2-mercaptoethanol (100 mmol/L), SDS (2%), and Tris.HCl (62.5 mmol/L), pH 7.8. Membranes were reprobed with the corresponding total antibody or GAPDH.

### ACC2 activity and Malonyl-CoA

ACC1 and ACC2 activity in gastrocnemius was measured by ^14^CO_2_ fixation into acid-stable products as recently described (Fullerton et al. 2013). Briefly, four 10 *μ*L replicates from each immunoprecipitated sample were incubated for 30 min at room temperature with 90 *μ*L reaction buffer containing acetyl-CoA (125 *μ*mol/L), [^14^C]-NaHCO_3_ (134 *μ*Ci/mL; equivalent to 2.5 mmol/L of NaHCO_3_) (Cat# NEC086H001MC; Perkin Elmer, Waltham, MA), HEPES (556 mmol/L), MgCl_2_ (111 mmol/L), MnCl_2_ (11 mmol/L), DTT (22 mmol/L), ATP (4 mmol/L), BSA (0.0075%), and +/− citrate (5 mmol/L). The reaction was stopped by the addition of 10 *μ*L of concentrated HCl and dried at 80°C. A second evaporation step was performed to reduce intersample variability by the addition of 50 *μ*L of 5 mol/L HCl and evaporated at 80°C. 200 *μ*L of H_2_O was added to the dried sample and [^14^C] radioactivity measured by liquid scintillation counting. Malonyl-CoA was measured in extracts from gastrocnemius muscle of fed mice using Ultra Performance Liquid Chromatography (UPLC) as recently described (Fullerton et al. 2013).

### Muscle glycogen and triglycerides

Glycogen content was determined in gastrocnemius muscle as glycosyl units after acid hydrolysis as previously described (Passonneau et al. 1967). Briefly, 10 mg w/w muscle was hydrolyzed in HCl (1 mol/L) at 98°C for 2 h and analyzed using an automatic analyzer (Hitachi automatic analyzer 912; Boehringer Mannheim, Ingelheim, Germany).

### Statistics

Unless otherwise noted, data were expressed as means ± standard error of the mean (SEM). Results were analyzed using Student’s *t-*test, paired *t-*test or analysis of variance (ANOVA) procedures where appropriate using GraphPad Prism software (La Jolla, CA). Significance was accepted at *P* < 0.05.

## Results

**Figure 1:**
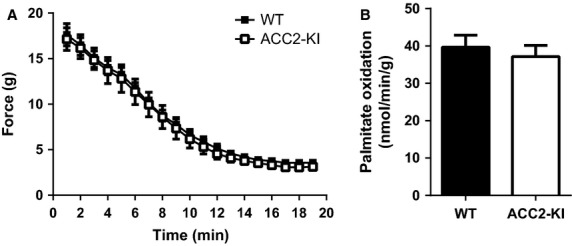
ACC2 S212 phosphorylation is not essential for regulating muscle performance or rates of fatty acid oxidation during muscle contractions. Force (A) and Palmitate oxidation (B) in extensor digitorum longus muscles from WT and ACC2 KI mice contracted ex vivo.

We first electrically stimulated *extensor digitorium longus* (EDL) muscles from WT and ACC2-KI mice ex vivo and found that there was no difference in fatigue curves (Fig.1A) or fatty acid oxidation (Fig.1B) between genotypes. Unfortunately given the small size of the EDL muscle it was not possible to measure ACC activity or malonyl-CoA following contractions. These data indicate that a lack of ACC2 S212 phosphorylation does not promote muscle fatigue or reduce rates of fatty acid oxidation during electrically stimulated contractions.

**Figure 2:**
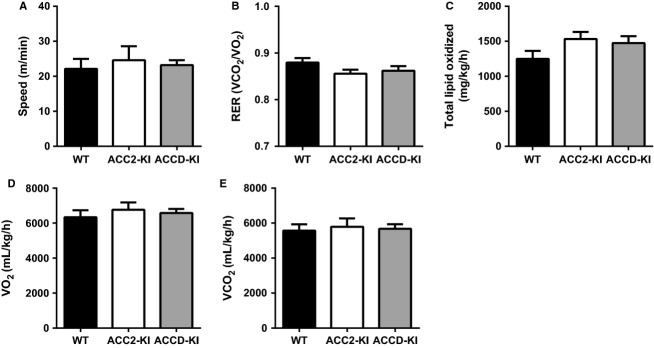
ACC2 S212 phosphorylation is not essential for maintaining exercise capacity and fatty acid oxidation during submaximal exercise. (A) Maximal running speed during a progressive treadmill running test in WT, ACC2-KI and ACCD-KI mice. (B) Average respiratory exchange ratio (RER), (C) calculated total lipid oxidized, (D) O_2_ consumption and (E) CO_2_ production in WT, ACC2-KI, and ACCD-KI during 60 min of submaximal treadmill running at the same relative intensity (65% each mouse’s max running capacity). Data are means ± SEM, *n*  = 6–7.

Given the artificial and intense nature of the ex vivo muscle contraction protocol we next examined treadmill running performance in WT and ACC2-KI mice and, to test for potential compensation by ACC1, ACCD-KI mice. We found that maximal treadmill running speed was not different between genotypes (Fig.2A). In subsequent experiments we collected respiratory gases during endurance treadmill running (60 min at same relative intensity [65% each individual mouse’s maximal running speed]) and found that average RER and calculated fatty acid oxidation (total lipids oxidized) for all mice were similar to WT (Fig.2B and C). Oxygen consumption (VO_2_) and carbon dioxide (CO_2_) production were not different from WT (Fig.2D and E). Circulating levels of glucose and lactate were not different between genotypes; however, NEFA levels were modestly reduced in ACC2 but not ACCD-KI mice (Table1). These data suggest that mice were exercising at the same relative intensity and the maintenance of fatty acid oxidation in ACC2-KI mice was not due to increased adipose lipolysis/substrate availability compared to WT mice. Consistent with similar rates of fatty acid oxidation during the treadmill exercise muscle glycogen levels at the completion of exercise were also not different between genotypes (Table1). Collectively, these data indicate that the phosphorylation of ACC1 Ser79 and ACC2 S212 is not required for maintaining endurance exercise capacity or whole-body rates of fatty acid oxidation during submaximal endurance exercise.

**Table 1:** Time course of serum metabolites during exercise.

Metabolite	Time (min)								
	0			30			60		
	WT	ACC2-KI	ACCD-KI	WT	ACC2-KI	ACCD-KI	WT	ACC2-KI	ACCD-KI
Glucose (mM)	8.40 ± 0.21	7.59 ± 0.22*	8.22 ± 0.36	11.85 ± 0.69#	10.04 ± 0.32#	11.90 ± 0.33#	11.30 ± 0.77#	10.06 ± 0.48#	11.17 ± 0.44#
NEFA (mEq/L)	0.97 ± 0.08	1.16 ± 0.07	1.13 ± 0.07	1.35 ± 0.09	1.20 ± 0.08	1.29 ± 0.08	1.55 ± 0.07	1.28 ± 0.06*	1.70 ± 0.13
Lactate (mM)	5.54 ± 0.32	5.49 ± 0.23	6.67 ± 0.37*	5.33 ± 0.31	5.26 ± 0.39	5.27 ± 0.34#	5.56 ± 0.60	5.19 ± 0.41	5.58 ± 0.18#
Glycogen (*μ*mol/g w/w)	11.33 ± 0.37	8.96 ± 0.25*	7.88 ± 0.69*	ND	ND	ND	4.68 ± 0.25	4.80 ± 0.20	4.43 ± 0.47

ND, Not determined.

Male ACC2-KI, ACCD-KI and WT mice were run on a treadmill at 65% maximal running speed and blood was collected at basal 0 min and 30 and 60 min for determination of serum glucose, non-esterified fatty acids (NEFA) and lactate as described under methods. Glycogen levels were measured in muscle at rest (as previously reported (12)) and following 60 min treadmill running. Data expressed as means ± SEM. *n* = 6–7 mice.

# *P *<* *0.05 compared to basal (time = 0 min), same genotype.

* *P *<* *0.05 compared to WT, same condition.

**Figure 3:**
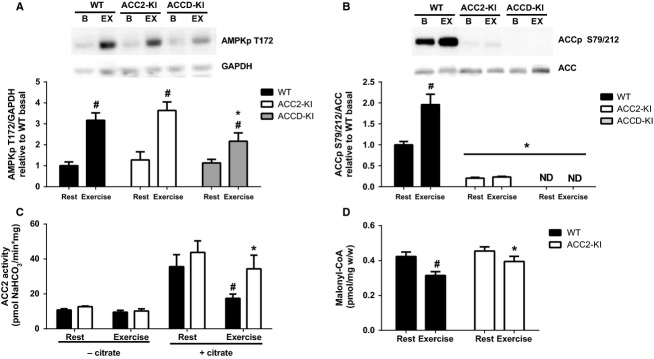
ACC2 S212 phosphorylation inhibits ACC2 activity and malonyl-CoA production during exercise. Mixed gastrocnemius muscle (A) AMPK T172 and (B) ACC2 S212 phosphorylation in ACC2-KI, ACCD-KI and WT mice at rest and following 60 min of submaximal treadmill running (65% maximal running capacity) relative to WT mice. (C) Mixed gastrocnemius muscle (C) ACC2 activity (+/− 5 mmol/L citrate) and (D) malonyl-CoA in WT and ACC2 KI mice at rest and following 60 min of submaximal treadmill running (65% maximal running capacity). Data are means ± SEM, *n*  = 6–11. ^#^*P* < 0.05 relative resting conditions, same genotype. *P < 0.05 compared to wild-type (WT) mice.

At the completion of the 60 min of treadmill exercise, as described above, muscles were collected from WT, ACC2-KI, and ACCD-KI mice. Treadmill exercise increased activating phosphorylation of AMPK T172 in WT mice and this was comparable in ACC2-KI mice, but slightly reduced in ACCD-KI mice (Fig.3A). The reason for this reduction in AMPK activating phosphorylation in ACCD-KI mice during treadmill running is not known. While exercise increased ACCS79/212 phosphorylation in WT mice this was not observed in ACC2-KI and ACCD-KI mice (Fig.3B). We subsequently measured ACC1 and ACC2 activity in muscles of ACC2-KI mice. We found that ACC1 activity in skeletal muscle was below the limits of detection of our assay in both WT and ACC2-KI mice despite the assay easily detecting ACC1-specific activity in liver samples (data not shown and Fullerton et al. 2013). These findings are consistent with previous reports that ACC1 activity is extremely low in skeletal muscle (Abu-Elheiga et al. 1995). Given the undetectable levels of muscle ACC1 activity further analysis was only conducted in ACC2-KI mice. Consistent with our previous findings (O’Neill et al. 2014) ACC2 activity tended to be upregulated in resting muscles of ACC2-KI mice compared to WT controls (Fig.3C). Importantly, we found that at the completion of exercise WT mice had reduced ACC2 activity but that this effect was blunted in ACC2-KI mice (Fig.3C). These data indicate that ACC2 S212 phosphorylation is essential for inhibiting ACC2 activity during treadmill exercise. We subsequently examined muscle malonyl-CoA in WT and ACC2-KI mice and found that consistent with changes in ACC2 activity, malonyl-CoA levels were reduced in WT but not ACC2-KI mice following the completion of exercise (Fig.3D). These data indicate that while ACC2 S212 phosphorylation is critical for regulating ACC2 activity and malonyl-CoA content during exercise, this is not required for regulating rates of fatty acid oxidation or exercise capacity during treadmill running.

**Figure 4:**
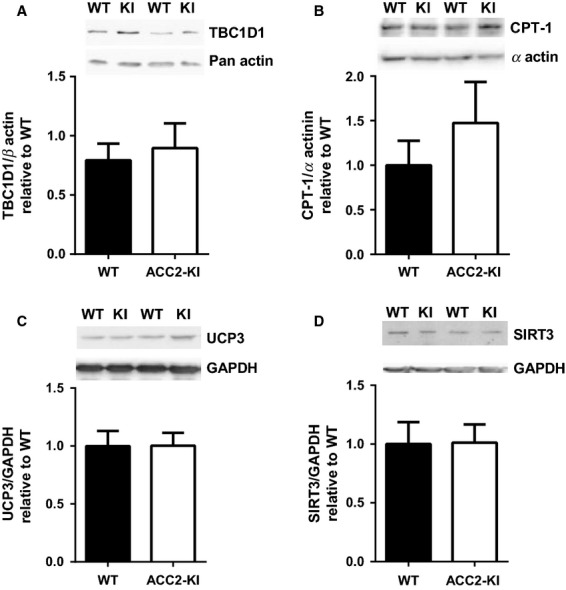
Expression of proteins involved in fatty acid oxidation in muscle. (A) TBC1D1 protein expression in quadriceps muscle of WT and ACC2 KI mice (*N* = 8–10). (B) CPT-1 expression in gastrocnemius muscle (*N* = 5–7). (C) UCP3 and (D) SIRT3 protein expression in quadriceps muscle (*N* = 8–10). Data are means ± SEM.

In an attempt to identify possible AMPK-ACC2-malonyl-CoA independent mechanisms regulating fatty acid oxidation during exercise we measured expression of proteins that have been implicated in the regulation of skeletal muscle fatty acid oxidation. Given many recent reports indicating a potentially important role for tre-2/USP6, BUB2, cdc16 domain family member 1 (TBC1D1) in inhibiting fatty acid oxidation (Chadt et al. 2008; Maher et al. 2014) and potential regulation through AMPK (O’Neill et al. 2011; Jeppesen et al. 2013; Fentz et al. 2015) we measured this protein and found no difference compared to WT littermates (Fig.4A). We also measured the expression of mitochondrial proteins implicated in the regulation of fatty acid oxidation (McGarry and Brown 1997; MacLellan et al. 2005; Hirschey et al. 2010) including CPT-1 (Fig.4B), UCP3 (Fig.4C), and SIRT3 (Fig.4D) and found that the expression of these proteins were not altered in muscle of ACC2-KI mice compared to WT controls. These data suggest that the maintenance of mitochondrial fatty acid oxidation in ACC2-KI mice during exercise, despite elevated ACC2 activity and malonyl-CoA, is not due to changes in the expression of TBC1D1, CPT1, UCP3, or SIRT3.

## Discussion

The AMPK-ACC2-malonyl-CoA signaling pathway has been implicated as the principal mechanism regulating fatty acid oxidation during exercise. This assumption has been based on studies using pharmacological agents such as AICAR and association studies during exercise showing activation of AMPK is associated with increases in ACC Ser79/212 phosphorylation and reductions in ACC activity and malonyl-CoA levels. Despite these strong associations previous studies in muscle AMPK deficient mouse models mice display normal (Dzamko et al. 2008; Steinberg et al. 2010; Jeppesen et al. 2013) or increased (O’Neill et al. 2011) rates of fatty acid oxidation during exercise/muscle contractions. However, a recent report in AMPK alpha muscle-specific null mice has found lower rates of fatty acid oxidation during exercise and muscle contractions, but these findings are complicated by the reduced mitochondrial function and lower rates of basal fatty acid oxidation in this mouse model (Fentz et al. 2015). In addition, in this study surprisingly there was also a substantial increase in ACC phosphorylation during contraction making it difficult to make conclusions about the importance of this pathway in controlling fatty acid oxidation during exercise/muscle contractions (Fentz et al. 2015). Therefore, the goal of this study was to examine the importance of the ACC-malonyl-CoA signalling axis in regulating fatty acid oxidation during exercise. We find that while ACC2 phosphorylation is essential for controlling muscle ACC2 activity and malonyl-CoA content, surprisingly this is not vital for regulating rates of fatty acid oxidation during exercise/muscle contractions.

In contrast to resting muscle where the AMPK-ACC-malonyl-CoA signaling pathway is vital for controlling rates of fatty acid oxidation (Fullerton et al. 2013; O’Neill et al. 2014) we find that ACC2-KI mice had normal rates of fatty acid oxidation when measured ex vivo during muscle contractions or in vivo during treadmill exercise. Importantly, despite high levels of malonyl-CoA, fatty acid flux into the mitochondria is not altered, indicating alternative pathways controlling fatty acid entry into the mitochondria may exist. Therefore, we assessed the expression of proteins implicated in the regulation of fatty acid oxidation in muscle (TBC1D1, CPT1*β*, UCP3, and SIRT3) and found their expression was not altered in ACC2-KI mice. Further genomic and proteomic analysis will need to be completed to examine whether there are alterations in other proteins capable of regulating fatty acid oxidation in muscle.

A second possibility for the maintenance of fatty acid oxidation in ACC2-KI mice may be that CPT-1*β* becomes insensitive to malonyl-CoA during exercise as previously suggested (Bezaire et al. 2004; Kerner et al. 2004; Holloway et al. 2006). Indeed our findings are consistent with a report by Smith et al. (Smith et al. 2012) who have established that the IC50 for malonyl-CoA inhibition of CPT-1*β* is much higher than would be predicted to allow for fatty acid oxidation during contractions. The reasons why muscle contractions but not AMPK activators (such as AICAR) may stimulate fatty acid oxidation independent of the ACC2-malonyl-CoA pathway is currently unclear. One hypothesis involves the idea that muscle contractions may activate a distinct set of kinases, which induce posttranslational modifications on CPT-1*β* that are important for desensitizing the enzyme to malonyl-CoA. For example Ca2+/calmodulin-dependent protein kinase and protein kinase A may be activated with exercise and could phosphorylate CPT-I to alter malonly-CoA sensitivity and/or CPT-I activity (Kerner et al. 2004; Sharma et al. 2010; Lundby et al. 2013; O’Neill et al. 2013). Future studies investigating this possibility may be important for understanding the mechanisms regulating fatty acid oxidation during exercise.

In addition to regulation by phosphorylation, lysine acetylation is an alternative mechanism that has recently been found to be important for regulating metabolism. Like phosphorylation, lysine acetylation can regulate enzyme activity of a range of proteins which are important for controlling fatty acid oxidation (reviewed in (Giralt and Villarroya 2012; Houtkooper et al. 2012). Indeed a recent study has found that genetic deletion of ACC results in alterations in acetylation profiles in the liver (Chow et al. 2014). Future studies investigating acetylation profiles in muscle of WT and ACC2-KI mice with and without exercise may reveal important new targets for controlling fatty acid oxidation.

One limitation of our study is that red and white muscle fiber types have differences in ACC activity, sensitivity to malonyl-CoA and oxidative capacity (Winder and Hardie 1996; Winder et al. 1997). We assessed palmitate oxidation in the glycolytic EDL muscle but due to the limited size of this muscle (∼10 mg) it was necessary to examine AMPK phosphorylation, ACC activity and malonyl-CoA content in mixed gastrocnemius muscle, which is more oxidative. While the mixed gastrocnemius muscle is more oxidative than EDL muscle it should be noted that it is reflective of the majority of the muscle mass in the mouse and as we also observed normal rates of fatty acid oxidation during whole body treadmill exercise our findings between these two muscle types appear to be consistent with the maintenance of fatty acid oxidation in the absence of ACC phosphorylation. A second limitation of our study was that it is possible that ACC KI mice have developed compensatory adaptations and phenotypes leading to masking or distortion of the acute role of this protein in regulating fatty acid oxidation (Barbaric et al. 2007). While in contrast to most genetic null models which remove the entire protein the mutations in our study were much more subtle and involved mutation of a single amino acid in either ACC1 and ACC2 proteins (Fullerton et al. 2013). However, we cannot rule out the possibility that compensatory upregulation of alternative pathways may have occurred masking the importance of this pathway in controlling fatty acid oxidation during exercise. Further proteomic studies may provide greater insight into the role of these compensatory pathways in regulating fatty acid oxidation.

In conclusion our studies indicate that during exercise/muscle contractions ACC2 phosphorylation is not vital for controlling rates of fatty acid oxidation. These studies highlight the important role of physiological redundancy in regulating metabolic flux during exercise and indicate that alternative pathways may be important. Future studies involving phosphoproteomic and acetylation profiling to identify ACC2-malonyl-CoA independent pathways will be necessary for understanding how fatty acid oxidation is regulated during exercise.

## Conflict of Interest

Authors do not have any conflict of interest to declare.
